# Electrical Stimulation Prevents Muscular Atrophy and the Decrease of Interleukin-6 in Paralyzed Muscles after Spinal Cord Injury in Rats

**DOI:** 10.1055/s-0044-1787767

**Published:** 2024-09-04

**Authors:** Nicolas A. M. M. Machado-Pereira, Patrícia S. do Nascimento, Gabriel R. de Freitas, Franciane Bobinski, Caroline C. do Espírito Santo, Jocemar Ilha

**Affiliations:** 1 Núcleo de Pesquisa em Lesão da Medula Espinal (NULEME), Departamento de Fisioterapia, Centro de Ciências da Saúde e do Esporte (CEFID), Universidade do Estado de Santa Catarina (UDESC), Florianópolis, SC, Brasil; 2 Departamento de Fisiologia e Farmacologia, Centro de Ciências da Saúde (CCS), Universidade Federal de Santa Maria (UFSM), Santa Maria, RS, Brasil; 3 Laboratório Experimental de Neurociências (LANEX), Universidade do Sul de Santa Catarina (UNISUL), Palhoça, SC, Brasil; 4 Instituto Internacional de Neurociências Edmond e Lily Safra (IIN-ELS), Macaíba, RN, Brasil

**Keywords:** electric stimulation therapy, interleukin-6, muscular atrophy, paralysis, rehabilitation, spinal cord injury

## Abstract

**Objective** To analyze the muscle trophism and expression of interleukin-6 in the biceps brachii muscle of rats with incomplete cervical spinal cord injury treated with neuromuscular electrical stimulation (NMES).

**Methods** Adult rats underwent C5-C7 spinal cord hemisection and a 5-week NMES protocol. Trophism of the biceps brachii was assessed using muscle weight/body weight ratio and histological analysis. Interleukin-6 expression from biceps brachii was measured using the enzyme-linked immunosorbent assay technique.

**Results** Preservation of the biceps brachii muscle trophism was found in the NMES treated group, along with prevention of the reduction of interleukin-6 levels.

**Conclusion** Spinal cord injury causes muscle atrophy and decreases interleukin-6 levels. These alterations are partially prevented by NMES. The results suggest a possible NMES action mechanism and underscore the clinical use of this therapeutic tool.

## Introduction

Spinal cord injury (SCI) is a clinical condition resulting mainly from trauma. 1 It disrupts the neural inputs between the spinal cord's upper and lower regions, impairing motor, sensory, and autonomous function below the injury level. Muscular paresis/paralysis is the most pronounced motor impairment after a SCI, being able to provoke an imbalance between anabolism and catabolism of several proteins, leading to protein breakdown or poor protein synthesis. 2

This protein breakdown may lead to several health concerns, such as hormonal changes, cardiovascular diseases, overweight and muscle loss. 3 4 Moreover, the protein imbalance causes changes in muscle fiber type, 5 promoting the reduction of fatigue resistance and reduced ability to generate force, 6 cross-sectional area and muscle weight due to loss of myofibrillar proteins, organelles, and cytoplasmic content. 7 8 9

The atrophy grade was pronounced in humans 1 to 4 months after SCI, with preferential type II muscle fibers atrophy. Additionally, a predominant type I fiber atrophy was seen in the later stage of the SCI (7–10 months). 10 11 Muscle atrophy and transformation of type I and II muscle fibers into type IIx leads to increased fatigue of these muscles. 12

Similarly, animal models have shown muscle atrophy after induced SCI. 13 14 In rats, 5 days after SCI, there is a decreased muscle fiber cross-sectional area of the soleus and extensor digitorum longus muscles. 11 The cross-sectional area continues to decrease for about 3 months after injury. 12 Additionally, there is reduced muscle strength 2 days after SCI, analyzed by grip test. 15

There is a range of strength and muscle mass gain treatments is available in clinical practice. Even so, neuromuscular electrical stimulation (NMES) is the treatment with the highest level of evidence for paralyzed and partially paralyzed muscles. Some studies have shown the benefits of electrically-induced involuntary muscle contraction in trophism and pointed out possible biological mechanisms underlying NMES in individuals with SCI 16 17 and animal models. 15 18 Recently, Freitas et al. 15 reported that in rats with SCI, the protein kinase B (Akt) pathway could be activated during involuntary muscle contraction by early application of NMES.

It is well established that the activation of Akt is the main pathway that leads to protein synthesis and hypertrophy after voluntary and functional electrical stimulation-induced muscle contraction, inducing muscle growth. 15 16 17 However, the involvement of the other molecules still needs to be investigated, such as the interleukin-6 (IL-6).

During and after active muscle contraction/voluntary exercise, the IL-6 is produced and released locally in muscle fiber and also released into the bloodstream, acting on muscle metabolism, glucose uptake, body heat production, promoting lipolysis and acting on the increase of muscle mass, 19 by activation of the phosphatidylinositol 3-kinase and possible activation of the Akt pathway contributing with muscle hypertrophy. 5

Within this context, IL-6 can be considered a stimulation factor of muscular trophism. However, it is unknown if involuntary electrically-induced repeated contractions of skeletal muscle can release IL-6 and prevent atrophy following a cervical SCI. Therefore, this study aims to analyze the muscular trophism and expression of IL-6 in the biceps brachii muscle of rats with cervical SCI treated early with NMES.

## Materials and Methods

Adult female Wistar rats aged 3 months were divided into the following groups: a) surgical simulation (sham; n = 5); b) untreated SCI (n = 5); and c) SCI treated with NMES (n = 5). The rats were anaesthetized with ketamine (87 mg/kg) and xylazine (12 mg/kg). Then, a laminectomy of the C5-C7 vertebrae and right-side spinal cord hemisection was performed using microscissors, except for the sham animals. 15 20

In this study, we had a mortality rate of 40% for all causes. There were 8 animals lost during the surgical procedure (32%) under anesthesia, mainly due to uncontrolled hemorrhages or respiratory failure, and 2 animals were lost during the 5-week intervention (8%) due to excessive sedation and respiratory fail. Procedures have been approved by the Committee for Care and Use of Animals of the local institution (protocol: PP00745).

After the surgical procedure, the animals were kept in clean, temperature-controlled boxes with water and food. They were evaluated twice daily to identify any suffering/pain or signs of urinary infection. The manual bladder emptying procedure was performed two or three times a day, and intraperitoneal antibiotics (Baytril 65 mg/kg) were administered to prevent and treat any possible infection in the first few days after the induced SCI until the animals recovered their bladder capacity.

For treatment protocol, the Neurodyn portable device (Ibramed, Amparo, SP, Brazil) was used with animals under 5% isoflurane inhalation anesthesia, starting 48 hours after the SCI. The protocol followed a regimen of 10 minutes per session, once a day, 5 days a week, with a total therapy time of 5 weeks. 15 A trichotomy of the anterosuperior part of the right forelimb was performed to place the electrodes on the skin, aiming for the biceps brachii muscle stimulation. The electrical stimulation parameters were asymmetric biphasic pulses, pulse duration of 150 µs and stimulation frequency of 50 Hz. The intensity was regularly adjusted to produce effective muscle contraction of visualizing complete elbow flexion.

### Evaluation of Muscular Trophism

After 5 weeks of treatment, the animals were euthanized, and the right biceps brachii muscle was dissected and weighed on a FA-2104 N precision scale –(Bioprecisa, Curitiba, PR, Brazil) to calculate muscle trophism measured by the ratio: muscle weight/animal weight. The muscle was sectioned longitudinally in the middle, and one part was frozen in liquid nitrogen and stored at -70°C for later biochemical analysis. The other part was separated for histological analysis.

### Histological Analysis

The muscle samples were immersed in 0.1 M phosphate buffered saline (PBS) containing 4% paraformaldehyde for 18 hours. Then, the muscle was immersed in 30% nitric acid for 48 hours and transferred to a 50% glycerol solution. 7 After that, four muscle fibers from each animal were placed on histological gelatinized slides, dehydrated in increasing series of ethanol concentrations (70, 90, and 100%, for 2 minutes each), immersed in xylol for 30 seconds and covered with Permount (Fisher Chemical, Pittsburgh, PA, USA) and coverslip.

Images of the fibers were captured with a Biological O400S 17MBO500RS (Opticam, São José dos Campos, SP, Brazil) optical microscope, with 1,000x magnification coupled with a HD212, 1920 × 1080p camera (AmScope, Irvine, CA, USA) and the AmScope software (AmScope), v3.7.9229. For the qualitative analysis, three images of each fiber were randomly selected from at least two animals per group. A blind evaluator performed the descriptive analysis of these images regarding the diameter of the fibers and the length of the sarcomeres.

### Biochemical Analysis

The IL-6 levels were measured using a sandwich enzyme-linked immunosorbent assay (ELISA) according to the manufacturer's instructions (DuoSet ELISA R&D Systems, Minneapolis, MN, USA). Muscle protein levels were estimated by interpolation from a standard curve using colorimetric measurements at 450 nm (wavelength correction = 540 nm) on an Apollo-8 LB 912 ELISA plate reader (Berthold Technologies GmbH & Co KG, Bad Wildbad, Germany). All results were expressed as pg/mg protein.

### Statistical Analysis

Results of the muscle weight/animal weight ratio and IL-6 levels were analyzed by one-way analysis of variance (ANOVA). The Tukey post hoc test was used for comparisons between groups. The significance level was 95% ( *p* < 0.05). All analyses were performed using the IBM SPSS Statistics for Windows (IBM Corp., Armonk, NY, USA) software, version 20.0.

## Results

### NMES Prevents Muscle Atrophy

**Fig. 1:**
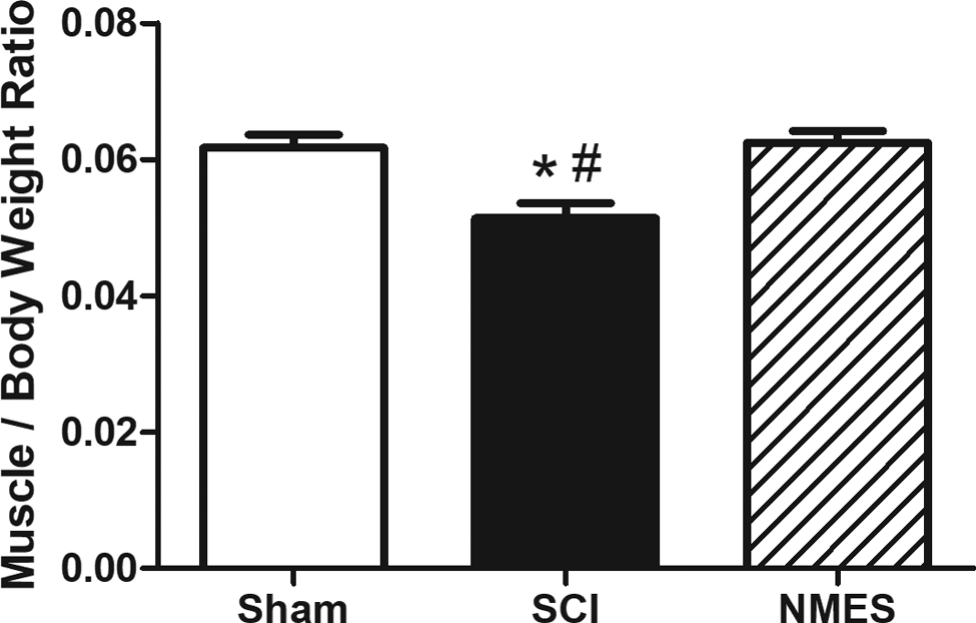
Effects of NMES for 5 weeks on the biceps brachii muscle trophism after 36 days of SCI. * *p* < 0.05 compared to the sham group; and # *p* < 0.05 compared to the treated group. Values are expressed as the mean and standard error of the mean.

The untreated SCI group showed a reduction in muscle trophism (0.051 ± 0.002 g) when compared to the sham group (0.061 ± 0.001 g; *p* < 0.05). However, the NMES treated group (0.062 ± 0.001 g) showed similar values to the sham group, both being higher than the untreated group ( *p* < 0.05; Fig. 1 ).

**Fig. 2:**
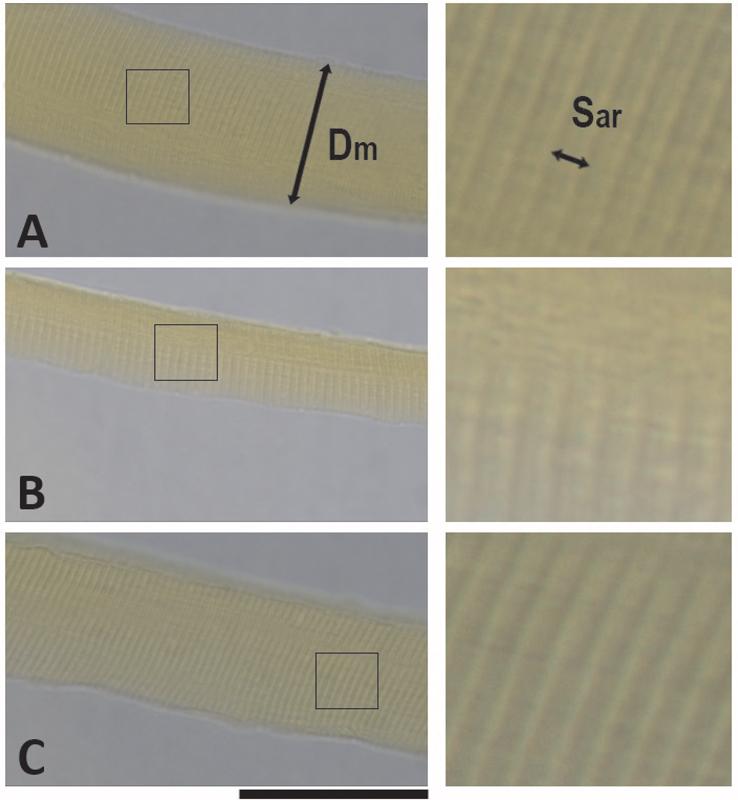
Histological images from the biceps brachii muscle. ( **A** ) Sham group, ( **B** ) untreated group, ( **C** ) NMES treated group, (Dm) diameter, (Sar) sarcomere. The black square is shown zoomed on the side. The black bar represents a space of 10µm.

Furthermore, according to the observational analysis of the muscle fibers under the light microscope, the transverse striations of all groups are presented in series and in an organized way. However, it is possible to verify that there is a decrease in the diameter and length of the sarcomeres of the muscle fibers in both untreated and NMES treated SCI groups compared to the sham group. On the other hand, there was a partial recovery in the NMES treated group, which is not as evident in the untreated group. It was observed that the diameter and length of the sarcomere in the NMES treated group were similar to the fibers of the sham group, evidencing a reduction in the loss of muscular trophism ( Fig. 2 ).

### NMES Prevents the Decrease of IL-6

**Fig. 3:**
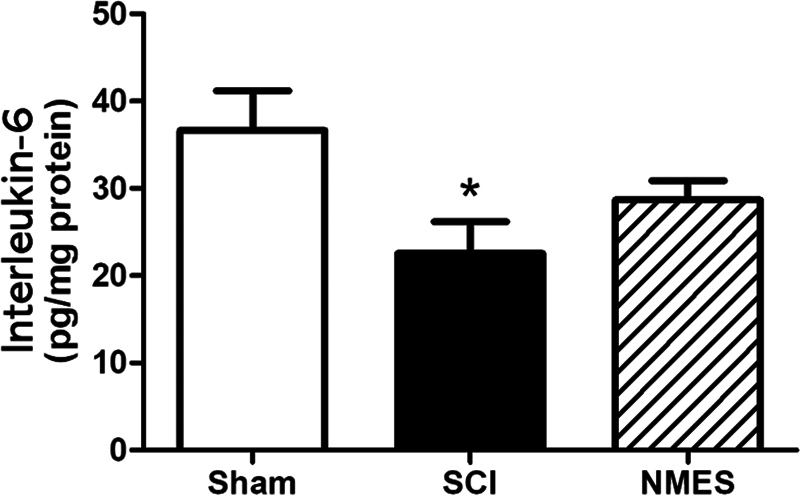
Effects of 5 weeks NMES on the expression levels of the Interleukin-6 from the biceps brachii muscle after 36 days of SCI. * *p* < 0.05 compared to the sham group. Values are expressed as the mean and standard error of the mean.

There was a decrease in expression of IL-6 in the untreated group (22.5 ± 3.6) compared to the sham group (36.6 ± 4.5; *p* < 0.05). However, the values of the NMES treated group (28.6 ± 2.2) were similar to both sham and untreated groups, evidencing partial preservation of the IL-6 expression in the NMES treated SCI group ( Fig. 3 ).

## Discussion

This study showed that applying NMES for 5 weeks prevents biceps brachii muscle atrophy after incomplete cervical SCI. Additionally, this therapy prevents loss in IL-6 levels, a potential stimulation factor of muscular trophism. Few studies assessed muscle trophism in rodents with SCI treated with NMES. 15 18 Additionally, no other study investigated the release and role of myokines, such as IL-6, in paralyzed muscles following NMES in a SCI model.

However, previous studies have shown the effects of NMES on muscle trophism after a complete or incomplete SCI model. Butezloff et al. 18 showed that the treated group (NMES for 4 weeks) had 54% more muscle mass in gastrocnemius than the untreated group. Additionally, this same study showed a decrease in the number and size of muscle fibers in rats with complete SCI by surgical transection of the spinal cord at the T10 level. Hayashi et al. 21 showed that 4 weeks of daily percutaneous electrical muscle stimulation promoted a significant improvement in motor function and an increase of brain-derived neurotrophic factor (BDNF) levels in both muscles and in the injury site in an incomplete SCI model, with contusion injury at the T9 level in rats. Moreover, de Freitas et al. 15 evidenced an increase of about 15% in muscle trophism of the biceps brachii muscle after 5 weeks of electrical stimulation in rats in a model of incomplete SCI by a spinal cord hemisection at the C5-C7 level in rats. Our data corroborate these results, showing about 17% more muscle mass in the treated injured animals than the untreated ones.

Our study also showed a reduction in the size of sarcomeres from both untreated and treated SCI groups. However, NMES partially recovered these characteristics once they were similar to sham animals. These findings indicate that electrically induced muscle contraction can prevent muscle atrophy caused by SCI and maintain the architectural features of muscle fibers.

Several studies have shown that IL-6 released during active exercise stimulates the activation of satellite cells, leading to increased muscle mass and consequent muscle hypertrophy. 6 22 Thus, during strength training, IL-6 is produced in large amounts at the local level, being considered an essential regulator in muscle satellite cell proliferation and myonuclear agglomeration, characterizing a biochemical-signaling pattern that stimulates muscle hypertrophy. 22 23

In our study, a decrease in local levels of IL-6 expression was found in paralyzed muscle after SCI, and involuntary muscle contraction elicited by electrostimulation for a short period could prevent this reduction. Bustamante et al. 24 showed that depolarization of skeletal muscle cells induces not only expression of IL-6 mediated by adenosine triphosphate (ATP) signaling but also cytokine secretion to the extracellular medium. Furthermore, this expression depends on an IP3-derived Ca signal, 2 acting as an early step to promote a positive IL-6 loop via the Janus kinase 2/signal transducer and activator of transcription 3 (JAK2/STAT3) pathway.

Additionally, several studies have reported that IL-6 can interact with the Akt pathway and regulate glycogen synthesis, thus acting in muscle trophism. 8 25 Moreover, IL-6 is released within the muscle with low glycogen levels. Therefore, muscle contraction and energy consumption are essential for its regulation, which arises by activating Ca ^2+^ nuclear factor of activated T cells (NFAT) and glycogen-P38 mitogen-activated protein kinase (MAPK) pathways.

It is known that type I muscle fibers are converted to type II after SCI. 12 This process has been reported within weeks in rats, and NMES can partially prevent this in the hindlimbs. 26 Our findings indicate that this prevention may have occurred in the forelimbs of rats with IL-6 participating in this process. However, some gaps remain, like the possibility of IL-6 being released by electrically induced muscle contraction inhibiting the catabolic pathway of trophism, whether there is an association between IL-6 levels and the number of type I muscle fibers, or whether another myosin participates in the process of maintaining muscle trophism and the characteristics of muscle fibers and their underlying mechanisms.

Liang et al. 27 showed that ex-vivo IL-6 released from skeletal muscles is type-specific, meaning treatment with a cytokine secretion stimulant cocktail induces a robust IL-6 release from the soleus, a typical slow oxidative muscle, and the same treatment was not able to cause a detectable release from the extender digitorum longus (a typical fast glycolytic muscle). However, as previously reported in SCI, 12 the type I muscle fibers are converted into type II, corroborating with the decreasing IL-6 in those fast glycolytic muscles. As expected, in the current study, we showed decreasing muscle IL-6 in SCI rats, and the NMES could prevent this decrease.

Additionally, other interleukins may be involved in muscle trophism beside IL-6, such as the leukemia inhibitory factor (LIF), a member of the IL–6 cytokine superfamily that has multiple biological functions. The LIF protein is secreted from human-cultured myotubes. When electrically stimulated, it causes satellite cell proliferation. It has been demonstrated that both IL-6 and LIF activate myotube mammalian target of rapamycin complex 1 (mTORC1) signaling in a time- and dose-dependent fashion. 28

## Conclusion

This study indicates that early NMES can prevent muscle atrophy and preserve the characteristics of muscle fibers in the biceps brachii after an incomplete SCI model. Moreover, the NMES partially maintained the reduction of IL-6 levels after injury in this muscle. We suggest that the NMES induced IL-6 release, possibly activating the satellite cells and increasing the phosphorylated Akt levels. However, further studies are required to elucidate the subsequent mechanisms of NMES on paralyzed muscles after SCI.
