# Supplementary material for: Electrical Stimulation Prevents Muscular Atrophy and the Decrease of Interleukin-6 in Paralyzed Muscles after Spinal Cord Injury in Rats
Source: Rev Bras Ortop (Sao Paulo). 2024 Sep 4;59(4):e526–31. doi: 10.1055/s-0044-1787767 (PMC11374404; doi:10.1055/s-0044-1787767)
Supplement: Related texts

# Research article: A estimulação elétrica previne a atrofia muscular e a diminuição da interleucina-6 em músculos paralisados após lesão da medula espinal em ratos

**Keywords:** atrofia muscular, interleucina-6, paralisia, reabilitação, terapia por estimulação elétrica, traumatismos da medula espinal

## Abstract

**Objetivo** Analisar o trofismo muscular e a de interleucina-6 no músculo bíceps braquial de ratas com lesão medular cervical incompleta tratados com estimulação elétrica neuromuscular (EENM).

**Métodos** Ratas adultas foram submetidas à hemissecção da medula espinal em C5-C7 e a um protocolo de EENM de 5 semanas. O trofismo do bíceps braquial foi avaliado pela relação peso muscular/peso corporal e análise histológica. A expressão de interleucina-6 no bíceps braquial foi medida usando ensaio de imunoabsorção enzimática.

**Resultados** O grupo tratado com EENM apresentou preservação do trofismo muscular, assim como prevenção da redução dos níveis de interleucina-6.

**Conclusão** A lesão da medula espinal causa atrofia muscular e diminui a expressão de interleucina-6. Essas alterações são parcialmente prevenidas pela EENM. Os resultados sugerem um possível mecanismo de ação da EENM e ressaltam o uso clínico desta ferramenta terapêutica.

## Introdução

A lesão da medula espinal (LME) é uma condição clínica resultante principalmente de traumas. 1 Ela interrompe as entradas neurais entre as regiões superior e inferior da medula espinal, prejudicando a função motora, sensorial e autônoma abaixo do nível da lesão. A paresia/paralisia muscular é o comprometimento motor mais pronunciado após a LME, podendo provocar um desequilíbrio entre o anabolismo e o catabolismo de diversas proteínas, levando à degradação ou má síntese proteica muscular. 2

Essa degradação de proteínas pode levar a diversos problemas de saúde, como alterações hormonais, doenças cardiovasculares, excesso de peso e perda muscular. 3 4 Além disso, o desequilíbrio proteico altera o tipo de fibra muscular, 5 promovendo a redução da resistência à fadiga, da capacidade de gerar força, 6 da área de secção transversal e do peso muscular devido à perda de proteínas miofibrilares, organelas e conteúdo citoplasmático. 7 8 9

A atrofia foi pronunciada em humanos 1 a 4 meses após a LME, afetando preferencialmente as fibras musculares do tipo II. Além disso, uma atrofia predominante das fibras do tipo I foi observada no estágio posterior da LME (7–10 meses). 10 11 Atrofia muscular e a transformação das fibras musculares do tipo I e II em tipo IIx aumentam a fatiga desses músculos. 12

Da mesma forma, modelos animais mostraram atrofia muscular após a LME induzida. 13 14 Em ratos, 5 dias após a LME, houve diminuição da área transversal da fibra muscular dos músculos sóleo e extensor longo dos dedos. 11 A área transversal continua a diminuir por cerca de 3 meses após a lesão. 12 Além disso, há redução da força muscular 2 dias após a LME ao teste de preensão. 15

Há uma gama de tratamentos para ganho de força e massa muscular na prática clínica. Ainda assim, a estimulação elétrica neuromuscular (EENM) é o tratamento com maior nível de evidência para músculos com paralisia parcial ou completa. Alguns estudos mostraram os benefícios da contração muscular involuntária induzida eletricamente no trofismo e apontaram possíveis mecanismos biológicos subjacentes à EENM em indivíduos com LME 16 17 e modelos animais. 15 18 Recentemente, de Freitas et al. 15 relataram que, em ratos com LME, a via da proteína quinase B (Akt) poderia ser ativada durante a contração muscular involuntária pela aplicação precoce de EENM.

Está bem estabelecido na literatura que a ativação de Akt é a principal via que leva à síntese e hipertrofia proteica após a contração muscular voluntária e induzida pela estimulação elétrica funcional, levando ao crescimento muscular. 15 16 17 No entanto, o envolvimento de outras moléculas, como a interleucina-6 (IL-6), ainda precisa ser investigado.

Durante e após a contração muscular ativa/exercício voluntário, há produção e liberação local de IL-6 na fibra muscular. A IL-6 também é liberada na corrente sanguínea, atuando no metabolismo muscular, captação de glicose, produção de calor corporal, promoção de lipólise e aumento da massa muscular 19 pela ativação da fosfatidilinositol 3-quinase e possível ativação da via Akt como contribuinte da hipertrofia muscular. 5

Dentro deste contexto, a IL-6 pode ser considerada um fator de estimulação do trofismo muscular. No entanto, não se sabe se contrações repetidas involuntárias do músculo esquelético por indução elétrica podem liberar IL-6 e prevenir a atrofia após uma LME cervical. Portanto, este estudo tem como objetivo analisar o trofismo muscular e os níveis de expressão de IL-6 no músculo bíceps braquiais de ratos com LME cervical submetidos ao tratamento precoce com EENM.

## Materiais e Métodos

Ratas adultas Wistar com 3 meses de idade foram divididos nos seguintes grupos: a) simulação cirúrgica ( *sham* ; n = 5); b) sem tratamento (n = 5); e c) LME tratada com EENM (n = 5). As ratas foram anestesiadas com cetamina (87 mg/kg) e xilazina (12 mg/kg). Em seguida, foi realizada laminectomia das vértebras C5-C7 e hemissecção medular direita com microtesoura, exceto nos animais do grupo *sham* . 15 20

No presente estudo, a taxa de mortalidade por todas as causas foi de 40%. Houve 8 mortes de animais durante o procedimento cirúrgico (32%) sob anestesia, principalmente devido a hemorragias não controladas ou insuficiência respiratória e 2 animais morreram durante o período de intervenção de 5 semanas (8%) devido à sedação excessiva e insuficiência respiratória. Os procedimentos foram aprovados pelo Comitê de Cuidado e Uso de Animais da instituição local (protocolo: PP00745).

Após o procedimento cirúrgico, os animais foram mantidos em caixas limpas, com temperatura controlada, água e ração. Os animais foram avaliados duas vezes ao dia para identificação de sofrimento/dor ou sinais de infecção urinária. O procedimento de esvaziamento vesical manual foi realizado duas ou três vezes ao dia e um antibiótico (Baytril, 65 mg/kg) foi administrado por via intraperitoneal para prevenir e tratar qualquer possível infecção nos primeiros dias após a indução da LME até que os animais recuperassem a capacidade vesical.

O protocolo de tratamento utilizou o aparelho portátil Neurodyn (Ibramed Amparo, SP, Brasil) com animais sob anestesia inalatória com isoflurano a 5%, a partir de 48 horas após a LME. O protocolo seguiu um regime de 10 minutos por sessão, uma vez ao dia, 5 dias por semana, com tempo total de terapia de 5 semanas. 15 Foi realizada tricotomia da parte anterosuperior do membro torácico direito para colocação dos eletrodos na pele, visando a estimulação do músculo bíceps braquial. Os parâmetros de estimulação elétrica foram pulsos bifásicos assimétricos, duração de pulso de 150 µs e frequência de estimulação de 50 Hz. A intensidade foi ajustada regularmente para produzir contração muscular eficaz e visualizar a flexão completa do cotovelo.

## Avaliação do Trofismo Muscular

Após 5 semanas de tratamento, os animais foram eutanasiados e o músculo bíceps braquial direito foi dissecado e pesado na balança de precisão FA-2104 N (Bioprecisa, Curitiba, PR, Brasil) para cálculo do trofismo muscular medido pela razão entre os pesos muscular e do animal. O músculo foi seccionado longitudinalmente no meio e uma parte foi congelada em nitrogênio líquido e armazenada a -70° C para posterior análise bioquímica. A outra parte foi separada para análise histológica.

## Análise Histológica

As amostras musculares foram imersas em salina tamponada com fosfato a 0,1 M contendo paraformaldeído a 4% por 18 horas. Em seguida, o músculo foi imerso em ácido nítrico 30% por 48 horas e transferido para solução de glicerol a 50%. 7 Quatro fibras musculares de cada animal foram colocadas em lâminas histológicas gelatinizadas, desidratadas em séries crescentes de concentrações de etanol (70, 90 e 100%, por 2 minutos cada), imersos em xilol por 30 segundos e cobertos com Permount (Fisher Chemical, Pittsburgh, PA, EUA) e lamínula.

As imagens das fibras foram capturadas com o microscópio óptico Biological O400S 17MBO500RS (Opticam, São José dos Campos, SP, Brasil) com ampliação de 1.000x acoplado a uma câmera HD212, 1920 × 1080p (AmScope, Irvine, CA, EUA) e software AmScope (AmScope), v3.7.9229. Para a análise qualitativa, três imagens de cada fibra foram selecionadas aleatoriamente de pelo menos dois animais por grupo. Um avaliador sem conhecimento dos grupos de animais realizou a análise descritiva dessas imagens quanto ao diâmetro das fibras e ao comprimento dos sarcômeros.

## Análise Bioquímica

Os níveis de IL-6 foram medidos utilizando um ensaio de imunoabsorção enzimática ( *enzyme-linked immunosorbent assay* , ELISA, em inglês) em sanduíche de acordo com as instruções do fabricante (DuoSet ELISA R&D Systems, Minneapolis, MN, EUA). Os níveis de proteína muscular foram estimados por interpolação a partir de uma curva-padrão obtida por medidas colorimétricas a 450 nm (correção de comprimento de onda = 540 nm) em um leitor de placas de ELISA Apollo-8 LB 912 (Berthold Technologies GmbH & Co KG, Bad Wildbad, Alemanha). Todos os resultados foram expressos como pg/mg de proteína.

## Análise Estatística

Os resultados da relação peso muscular/peso do animal e dos níveis de IL-6 foram analisados por análise de variância ( *analysis of variance* , ANOVA, em inglês) unidirecional. O teste post hoc de Tukey foi utilizado para comparações entre grupos. O nível de significância foi de 95% ( *p* < 0,05). Todas as análises foram realizadas no programa IBM SPSS Statistics for Windows (IBM Corp., Armonk, NY, EUA), versão 20.0.

## Resultados

## Prevenção da Atrofia Muscular por EENM

O grupo LME não tratado apresentou redução no trofismo muscular (0,051 ± 0,002 g) em comparação ao grupo *sham* (0,061 ± 0,001 g; *p* < 0,05). Porém, o grupo tratado com EENM (0,062 ± 0,001 g) apresentou valores semelhantes ao grupo *sham* , ambos sendo superiores ao grupo não tratado ( *p* < 0,05; Fig. 1 ).

**Fig. 1:** Efeitos da estimulação elétrica neuromuscular (EENM) por 5 semanas no trofismo do músculo bíceps braquial após 36 dias da lesão da medula espinal (LME). * *p* < 0,05 em comparação ao grupo de simulação cirúrgica ( *sham* ); e # *p* < 0,05 em comparação ao grupo tratado. Os valores são expressos como média e erro-padrão da média.

Além disso, de acordo com a análise observacional das fibras musculares à microscopia óptica, as estrias transversais de todos os grupos estavam em série e organizadas. Porém, era possível verificar que uma diminuição no diâmetro e comprimento dos sarcômeros das fibras musculares tanto no grupo LME não tratado quanto no grupo tratado com EENM em comparação ao grupo *sham* . Por outro lado, houve uma recuperação parcial no grupo tratado, o que não é tão evidente no grupo não tratado. Observou-se que o diâmetro e o comprimento do sarcômero no grupo tratado foram semelhantes aos observados nas fibras do grupo *sham* , evidenciando a redução na perda de trofismo muscular ( Fig. 2 ).

**Fig. 2:** Imagens histológicas do músculo bíceps braquial. ( **A** ) Grupo de simulação cirúrgica ( *sham* ), ( **B** ) grupo não tratado, ( **C** ) grupo tratado com EENM, (Dm) diâmetro, (Sar) sarcômero. O quadrado preto mostra a imagem em maior aumento. A barra preta representa um espaço de 10 µm.

## Prevenção da Redução de IL-6 por EENM

Houve diminuição na expressão de IL-6 no grupo não tratado (22,5 ± 3,6) em comparação ao grupo *sham* (36,6 ± 4,5; *p* < 0,05). No entanto, os valores do grupo tratado com EENM (28,6 ± 2,2) foram semelhantes aos grupos *sham* e não tratado, evidenciando preservação parcial da expressão de IL-6 no grupo submetido ao tratamento (EENM) ( Fig. 3 ).

**Fig. 3:** Efeitos da EENM por 5 semanas nos níveis de expressão de interleucina-6 do músculo bíceps braquial após 36 dias de lesão da medula espinal (LME). * *p* < 0,05 em comparação ao grupo de simulação cirúrgica ( *sham* ). Os valores são expressos como média e erro-padrão da média.

## Discussão

Este estudo mostrou que a aplicação da EENM por 5 semanas previne a atrofia do músculo bíceps braquial após a LME cervical incompleta. Além disso, esse tratamento previne a perda da expressão de IL-6, um possível fator de estimulação do trofismo muscular. Poucos estudos avaliaram o trofismo muscular em roedores com LME tratados com EENM. 15 18 Além disso, nenhum outro estudo investigou a liberação e o papel de miocinas, como a IL-6, em músculos paralisados após EENM em um modelo de LME.

No entanto, estudos anteriores mostraram os efeitos da EENM no trofismo muscular em um modelo de LME completo ou incompleto. Butezloff et al. 18 mostraram que o grupo tratado (EENM por 4 semanas) apresentou 54% mais massa muscular no gastrocnêmio que o grupo não tratado. Além disso, este mesmo estudo mostrou uma diminuição no número e tamanho das fibras musculares em ratos com LME completa por transecção cirúrgica da medula espinal no nível de T10. Hayashi et al. 21 mostraram que 4 semanas de estimulação elétrica muscular percutânea diária promoveram uma melhora significativa na função motora e um aumento nos níveis do fator neurotrófico derivado do cérebro ( *brain-derived neurotrophic fator* , BDNF, em inglês) em ambos os músculos e no sítio da lesão em um modelo de LME incompleta, com lesão por contusão no nível T9 em ratos. Além disso, de Freitas et al. 15 evidenciaram um aumento de cerca de 15% no trofismo muscular do músculo bíceps braquial após 5 semanas de estimulação elétrica em ratos em um modelo de LME incompleta por hemissecção da medula espinal no nível C5-C7 em ratos. Nossos dados corroboram esses resultados, mostrando um aumento de cerca de 17% de massa muscular nos animais com LME tratados em comparação aos não tratados.

Nosso estudo também mostrou uma redução no tamanho dos sarcômeros nos grupos de LME com e sem tratamento. No entanto, a EENM recuperou parcialmente essas características, uma vez que os resultados foram semelhantes aos dos animais do grupo *sham* . Esses achados indicam que a contração muscular induzida eletricamente pode prevenir a atrofia muscular causada pela LME e manter as características arquitetônicas das fibras musculares.

Diversos estudos demonstraram que a IL-6 liberada durante o exercício ativo estimula a ativação de células-satélites, levando ao aumento da massa muscular e consequente hipertrofia muscular. 6 22 Assim, durante o treinamento de força, a IL-6 é produzida em grandes quantidades em nível local, sendo considerada um regulador essencial na proliferação de células-satélites musculares e na aglomeração mionuclear, caracterizando um padrão de sinalização bioquímica que estimula a hipertrofia muscular. 22 23

Nosso estudo detectou uma diminuição nos níveis locais de expressão de IL-6 no músculo paralisado após a LME e que a contração muscular involuntária provocada pela eletroestimulação por um curto período poderia prevenir essa redução. Bustamante et al. 24 mostraram que a despolarização das células musculares esqueléticas induz não apenas a expressão de IL-6 mediada pela sinalização de trifosfato de adenosina ( *adenosine triphosphate* , ATP, em inglês), mas também a secreção de citocinas para o meio extracelular. Além disso, essa expressão depende de um sinal de Ca ^2^ derivado de IP3, atuando como um passo inicial para promover uma alça positiva de IL-6 através da via Janus quinase 2/transdutor de sinal e ativador de transcrição 3 ( *Janus kinase 2/signal transducer and activator of transcription 3* , JAK2/STAT3, em inglês).

Ademais, vários estudos relataram que a IL-6 pode interagir com a via Akt e regular a síntese de glicogênio, atuando no trofismo muscular. 8 25 Além disso, IL-6 também é liberada dentro do músculo com baixos níveis de glicogênio. Portanto, a contração muscular e o consumo de energia são essenciais para sua regulação, derivada da ativação das vias Ca ^2+^ -fator nuclear de células T ativadas ( *nuclear factor of activated T cells* , NFAT, em inglês) e glicogênio-P38 proteína quinase ativada por mitógeno ( *mitogen-activated protein kinase* , MAPK, em inglês).

Sabe-se que as fibras musculares do tipo I são convertidas em tipo II após a LME. 12 Esse processo foi relatado em semanas em ratos e a EENM pode prevenir isso parcialmente nos membros posteriores. 26 Nossos achados indicam que essa prevenção pode ter ocorrido nos membros anteriores de ratos com participação de IL-6. No entanto, ainda há algumas lacunas, como a possibilidade de a IL-6 ser liberada pela contração muscular induzida eletricamente e inibindo a via catabólica do trofismo, se existe uma associação entre os níveis de IL-6 e o número de fibras musculares do tipo I, ou se outra miosina participa do processo de manutenção do trofismo muscular e das características das fibras musculares e seus mecanismos subjacentes.

Liang et al. 27 mostraram que a IL-6 liberada *ex vivo* dos músculos esqueléticos é específica do tipo de fibra muscular, ou seja, o tratamento com um coquetel estimulante da secreção de citocinas induz uma liberação robusta de IL-6 pelo sóleo, um músculo oxidativo lento típico, mas o mesmo tratamento não foi capaz de causar uma liberação detectável do extensor longo dos dedos (um típico músculo glicolítico rápido). Porém, como relatado anteriormente na LME, 12 as fibras musculares do tipo I são convertidas em tipo II, corroborando com a diminuição da IL-6 nesses músculos glicolíticos rápidos. Como esperado, no presente estudo, mostramos diminuição da IL-6 muscular em ratos com LME, e a EENM poderia prevenir essa diminuição.

Além disso, outras interleucinas além da IL-6 podem estar envolvidas no trofismo muscular, como o fator inibitório da leucemia (LIF), um membro da superfamília de citocinas IL-6 com múltiplas funções biológicas. A proteína LIF é secretada por miotubos humanos em cultura. Após a estimulação elétrica, ela causa a proliferação de células-satélites. Foi demonstrado que tanto a IL-6 quanto a LIF ativam a sinalização do alvo mecanicista do complexo 1 de rapamicina ( *mammalian target of rapamycin complex 1* , mTORC1, em inglês) do miotubo de forma dependente do tempo e da dose. 28

## Conclusão

Este estudo indica que a EENM precoce pode prevenir a atrofia muscular e preservar as características das fibras musculares do bíceps braquial após um modelo de LME incompleta. Além disso, a EENM manteve parcialmente a redução do nível de IL-6 após a lesão neste músculo. Nós sugerimos que a EENM induziu a liberação de IL-6 no músculo, possivelmente ativando as células-satélites e aumentando os níveis de Akt fosforilada. No entanto, mais estudos são necessários para elucidar os mecanismos subsequentes da EENM nos músculos paralisados após a LME.
